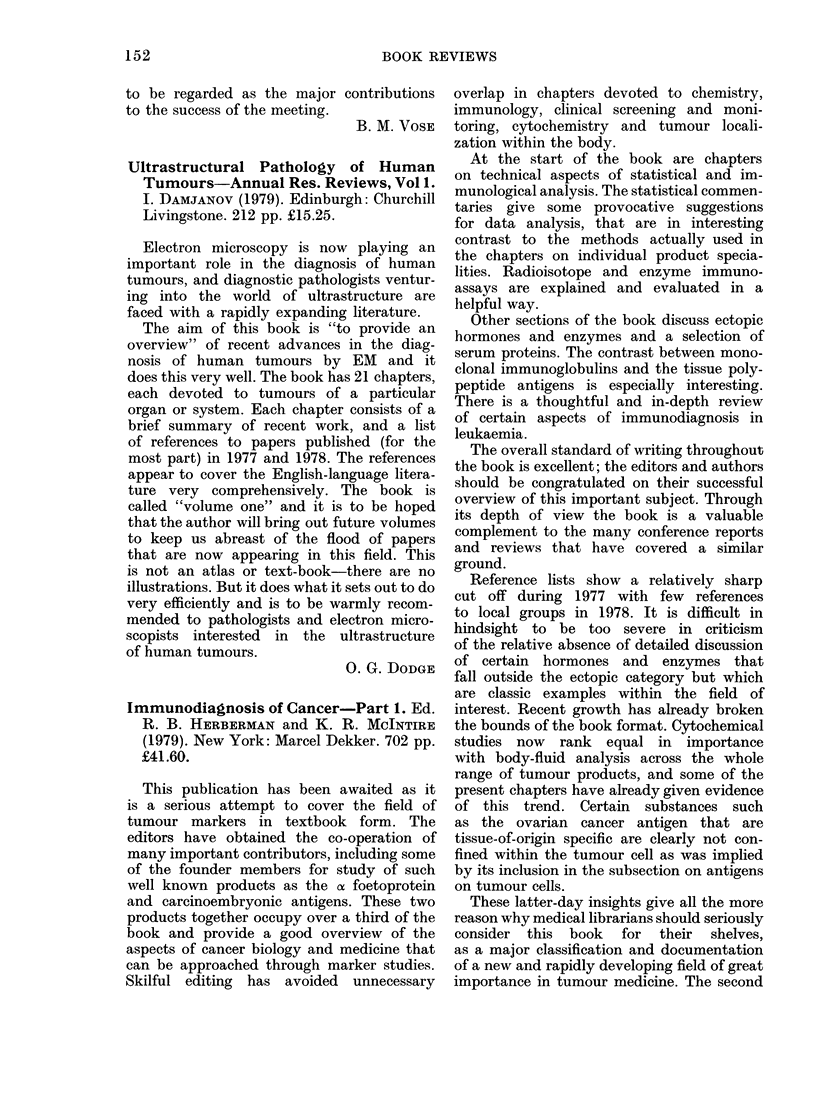# Ultrastructural Pathology of Human Tumours—Annual Res. Reviews, Vol 1

**Published:** 1980-01

**Authors:** O. G. Dodge


**Ultrastructural Pathology of Human Tumours—Annual Res. Reviews, Vol 1.** I. DAMJANOV (1979). Edinburgh: Churchill Livingstone. 212 pp. £15.25.

Electron microscopy is now playing an important role in the diagnosis of human tumours, and diagnostic pathologists venturing into the world of ultrastructure are faced with a rapidly expanding literature.

The aim of this book is "to provide an overview" of recent advances in the diagnosis of human tumours by EM and it does this very well. The book has 21 chapters, each devoted to tumours of a particular organ or system. Each chapter consists of a brief summary of recent work, and a list of references to papers published (for the most part) in 1977 and 1978. The references appear to cover the English-language literature very comprehensively. The book is called "volume one" and it is to be hoped that the author will bring out future volumes to keep us abreast of the flood of papers that are now appearing in this field. This is not an atlas or text-book—there are no illustrations. But it does what it sets out to do very efficiently and is to be warmly recommended to pathologists and electron microscopists interested in the ultrastructure of human tumours.

O. G. DODGE